# Effectiveness and acceptability of myoinositol in prevention of gestational diabetes mellitus: A protocol for systematic review and meta-analysis

**DOI:** 10.1097/MD.0000000000025673

**Published:** 2021-04-30

**Authors:** Li Wang, Hong Cheng, Xue Wang, Linmei Zheng

**Affiliations:** a Department of Obstetrics, Hainan People's Hospital; b Department of Obstetrics, Haikou Hospital of the Maternal and Child Health; c Department of Emergency, Hainan People's Hospital, Hainan, China.

**Keywords:** Gestational diabetes mellitus, myoinositol, meta-analysis, review, protocol

## Abstract

**Background:**

The existing meta-analyses and randomized studies on myoinositol are of poor quality, with small sample sizes, and involve a homogeneous population. The general applicability of these findings to the National Health Service is unclear. We thus conduct this new high-quality systematic review and meta-analysis to assess the efficacy and safety of myoinositol in pregnant woman.

**Methods:**

The study protocol will be developed and executed in compliance with the Preferred Reporting Items for Systematic Reviews and Meta-analyses statement. All of the following inclusion criteria in the PICOS order will be met by the studies included in our meta-analysis:

1. population: pregnant woman without gestational diabetes mellitus (GDM);
2. intervention: group with myoinositol;
3. comparison intervention: group without myoinositol;
4. outcome measures: at least one of the following outcome measures should to be reported: rate of GDM, offspring birthweight, fasting glucose, oral glucose tolerance test, and the side effects associated with the myoinositol; and
5. study design: English randomized trials.

The following electronic databases will be searched: PubMed, Scopus, EMBASE, and Cochrane Library databases. The Cochrane risk of bias tool will be used to evaluate the risk of bias of the included randomized trials by 2 independent reviewers.

**Results:**

We will perform a meta-analysis using standard techniques for the outcomes.

**Conclusions:**

It was hypothesized that myoinositol supplementation could increase the action of endogenous insulin and prevent GDM and its complications.

**Trial registration:**

10.17605/OSF.IO/9W8DV.

## 1 Introduction

Gestational diabetes mellitus (GDM), defined as impaired glucose tolerance that begins or is first recognized during pregnancy, is characterized by increased insulin resistance.^[1,2]^ The prevalence of this complication is increasing worldwide. The onset of GDM is associated with several risk factors, such as ethnic background, family history, high maternal age, increased body mass index, or a previous history of GDM.^[3]^ Although the reported incidence of GDM indicates that the pregnancy rate for the disease has reached 10%, the incidence is expected to be even higher, almost doubling, according to the newly proposed criteria for the diagnosis of GDM.^[4]^

Myoinositol is a polyol that, due to its differential isomerization, can appear in 9 stereoisomerisms depending on the distribution of its 6 hydroxyl groups. It is thought to be a pseudovitamin and belongs to the B vitamin complex; however, the definition of inositol as a vitamin is not entirely correct, because the body produces sufficient amounts of it from glucose.^[5–7]^ It occurs in the liver, kidneys, and brain. Myoinositol has been described as a second messenger system that may have an insulin-like effect on metabolic enzymes, thereby increasing insulin sensitivity.^[8]^ It has also been reported to have beneficial effects in preventing GDM in some randomized trials and meta-analyses.^[9–12]^

Nevertheless, the existing meta-analyses and randomized studies on myoinositol are of poor quality, with small sample sizes, and involve a homogeneous population. The general applicability of these findings to the National Health Service is unclear. We thus conduct this new high-quality systematic review and meta-analysis to assess the efficacy and safety of myoinositol in pregnant woman. It was hypothesized that myoinositol supplementation could increase the action of endogenous insulin and prevent GDM and its complications.

## 2 Materials and methods

### 2.1 Eligibility criteria

The study protocol will be developed and executed in compliance with the Preferred Reporting Items for Systematic Reviews and Meta-analyses statement. All of the following inclusion criteria in the PICOS order will be met by the studies included in our meta-analysis:

1. population: pregnant woman without GDM;
2. intervention: group with myoinositol;
3. comparison intervention: group without myoinositol;
4. outcome measures: at least one of the following outcome measures should to be reported: rate of GDM, offspring birthweight, fasting glucose, oral glucose tolerance test, and the side effects associated with the myoinositol; and
5. study design: English randomized trials.

Articles with no assessment of the outcomes mentioned above or no comparison of 2 groups will not be included in this meta-analysis. Duplicate reports and conference abstracts will be excluded. Retrospective trials, case reports, biochemical trials, letters, and reviews will also be eliminated. Two independent authors will screen the titles and abstracts of the potentially relevant studies to determine their eligibility based on the criteria. Disagreements will be resolved through a discussion with a third review author.

### 2.2 Search strategy

The following electronic databases will be searched: PubMed, Scopus, EMBASE, and Cochrane Library databases. There will be English language restriction. We developed a search strategy using a combination of keywords and medical subject headings (MeSH)/EMTREE terms, and the following expressions will be used: (Myoinositol or inositol) and (adductor canal block or saphenous nerve block or peripheral nerve block) and (gestational diabetes mellitus or pregnant or GDM) and (blind or random). The reference lists of the included studies will also be checked for additional studies that are not identified in the database search. This study will be reported in line with Assessing the Methodological Quality of Systematic Reviews guidelines. The systematic review protocol has been registered on Open Science Framework registries. Ethical approval is not necessary because the present meta-analysis will be performed based on previously published studies.

### 2.3 Data extraction

Two independent authors extract the following descriptive raw information from the selected studies: study characteristics such as author, publication year, study design; patient demographic details such as patients’ number, average age, body mass index, and gender ratio. Outcome measures include rate of GDM, offspring birthweight, fasting glucose, oral glucose tolerance test, and the side effects associated with the myoinositol. Where disagreement in the collection of data occurs, this is resolved through discussion. If the data are missing or can not be extracted directly, we will contact the corresponding authors to ensure that the information integrated. If necessary, we will abandon the extraction of incomplete data.

### 2.4 Statistical analysis

According to the basic characteristics of the included studies, the meta-analysis will be performed using Review Manager version 5.3 provided by the Cochrane Collaboration. Given the characteristics of the extracted data in the review, continuous outcomes will be expressed as the mean differences with 95% confidence intervals. Differences in categorical variables will be expressed as risk ratio values and 95% confidence intervals. Heterogeneity will be assessed by means of *I*^2^ statistics. *I*^2^ ≥ 50% represent high heterogeneity. A standardized mean difference will be used when the studies included in the meta-analysis assess the outcome based on different scales. Initially, a fixed-effect model will be used to compare the outcomes, unless the heterogeneity tests indicate that the *I*^2^ statistic ≥50% and substantial heterogeneity existed between studies; in this case, the reasons for this heterogeneity will be searched for and a random-effect model will be used for comparison. The publication bias will be assessed by using funnel plots diagram. The funnel plot asymmetry will be evaluated by an Egger linear regression test to reveal any possible publication bias. Sensitivity analyses will be undertaken to determine the potential source of heterogeneity when significant.

### 2.5 Quality assessment

The Cochrane risk of bias tool will be used to evaluate the risk of bias of the included randomized trials by 2 independent reviewers. Randomized trial quality will be assessed using the following 7 items: random sequence generation, allocation concealment, blinding of participants and personnel, blinding of outcome assessment, incomplete outcome data, selective reporting, and other bias. Kappa values will be used to measure the degree of agreement between the 2 reviewers and are rated as follows: fair, 0.40–0.59; good, 0.60–0.74; and excellent, >0.75. Any controversy will be resolved by discussion with a third author to reach a final consensus.

## 3 Discussion

In last years, different studies have investigated the preventive effects of myoinositol supplementation for GDM. Myoinositol has been reported to have beneficial effects in preventing GDM in some randomized trials and meta-analyses.^[9–12]^ Nevertheless, the existing meta-analyses and randomized studies on myoinositol are of poor quality, with small sample sizes, and involve a homogeneous population. The general applicability of these findings to the National Health Service is unclear. We thus conduct this new high-quality systematic review and meta-analysis to assess the efficacy and safety of myoinositol in pregnant woman. It was hypothesized that myoinositol supplementation could increase the action of endogenous insulin and prevent GDM and its complications. We foresee several potential limitations with this systematic review: heterogeneity of clinical outcomes, substandard quality of existing studies, which are the focus of our project. Therefore, we will present our findings using descriptive methods, if necessary. Our hope is that the dissemination of this protocol will allow us to obtain feedback and constructive criticism of the methods before our study is conducted.

## Author contributions

**Conceptualization:** Xue Wang.

**Data curation:** Li Wang, Hong Cheng.

**Formal analysis:** Hong Cheng.

**Funding acquisition:** Li Wang, Linmei Zheng.

**Investigation:** Li Wang, Hong Cheng.

**Methodology:** Xue Wang.

**Project administration:** Linmei Zheng.

**Resources:** Linmei Zheng.

**Software:** Li Wang, Hong Cheng.

**Supervision:** Linmei Zheng.

**Validation:** Xue Wang.

**Visualization:** Hong Cheng, Xue Wang.

**Writing – original draft:** Li Wang, Hong Cheng.

**Writing – review & editing:** Xue Wang, Linmei Zheng.
